# Editorial: Response and Resistance in Castration-Resistant Prostate Cancer

**DOI:** 10.3389/fonc.2020.607298

**Published:** 2020-10-23

**Authors:** Renée de Leeuw, Qi Cao, Hung-Ming Lam

**Affiliations:** ^1^Department of Pathology, University of Illinois at Chicago, Chicago, IL, United States; ^2^Department of Urology and Robert H. Lurie Comprehensive Cancer Center, Northwestern University Feinberg School of Medicine, Chicago, IL, United States; ^3^Department of Urology, University of Washington School of Medicine, Seattle, WA, United States

**Keywords:** neuroendocrine prostate cancer, castration-resistant prostate cancer, androgen deprivation therapy (ADT), new therapy, treatment resistance

Androgen-deprivation therapy (ADT), often coupled with androgen receptor (AR)-targeted therapies, has been the mainstay therapy for patients with advanced prostate cancer (PC) for over seven decades (1). Inevitably, the disease will progress to castration-resistant prostate cancer (CRPC) in a median of 3–4 years. Second-generation AR-targeted therapies (abiraterone, enzalutamide) and chemotherapy (docetaxel, cabazitaxel) are effective in some patients. However, responsive tumors eventually develop resistance. In this Research Topic, we have organized a collection of opinion, review, and original research articles that discuss the history, biology, and therapeutic opportunities in CRPC.

PC can develop ADT resistance in multiple ways, such as CRPC or development of neuroendocrine PC (NEPC) (2, 3). Wang, Gao et al. demonstrated that Lysine specific demethylase 1 (LSD1) activated PI3K/AKT pathways in the absence of androgen and triggered AR transcriptional activity that drives PC initiation and progression to CRPC. They report that LSD1 transcriptionally regulates the expression of PI3K regulatory subunit, p85, and propose that this may occur through epigenetic reprogramming of the enhancer landscape in PC. This study suggests that LSD1 has dual functions in promoting PC development by enhancing AR signaling through its coactivator function and activating PI3K/AKT signaling.

Elevated expression of neuroendocrine markers and increased angiogenesis are the two hallmarks of NEPC. To date, the direct molecular links between these phenotypes of NEPC and their mechanisms remain largely unclear. Wang, Zhao et al. summarize the literature on proteins reported to regulate both phenotypes of NEPC, which include AURKA/B, CHGA, CREB1, EZH2, FOXA2, GRK3, HIF1, IL-6, MYCN, ONECUT2, p53, RET, and RB1. This review highlights the current efforts to target these proteins and potential therapeutic options to treat NEPC. Lee et al. discuss the potential role of a neuronal-specific RNA splicing factor, Ser/Arg repetitive matrix-4 (SRRM4), in reprogramming the transcriptome to facilitate the differentiation, proliferation, and survival of cells to establish a NEPC phenotype. This review explores the roles of SRRM4 with other pathways in driving a NEPC program as a coping mechanism for therapy resistance and defines potential therapeutic approaches targeting SRRM4 for treating NEPC.

To better understand cancer progression and therapy resistance, it is critical to investigate not only cancer-specific molecular alterations, but also global burden of genetic aberrations, genetic and non-genetic heterogeneity and dynamicity, and the cancer “ecosystem.” Increased mutational burden does not appear to drive treatment-emergent NEPC. Ryan and Bose assessed published PC cohorts for global burden of mutations and chromosomal structural variants across tumor stages, rather than individual aberrations. As anticipated, overall mutational burden, structural variants, copy number alterations all independently increase as disease progresses. However, this relationship does not appear to be linear. This review stresses that there is complexity beyond genomic alteration type, quantity, and clonality in the ability to predict cancer progression.

Jolly et al. propose alternate mechanisms that can layer on to genomic events to promote therapeutic resistance, including phenotypic plasticity and variability in genetically identical cells. This concept is better known from bacterial biology, where persisters survive antibiotic treatment and give rise to genetically similar populations. The authors build a compelling case for viewing heterogeneity beyond clonality, regarding tumors as an ecosystem that facilitates cellular phenotypic switching, allowing the tumor to withstand therapeutic assaults. Clinically, this idea of non-genetic, phenotypic plasticity in cancer is supported by positive responses after a “drug holiday.” To further illustrate the relevance of non-genomic heterogeneity and plasticity, this review provides examples of therapeutic resistance that cannot be solely explained by clonal evolution.

CRPC generally shows sustained AR signaling which can be therapeutically exploited. Lam and Corey discuss the potential for paradoxically introducing androgen (i.e., testosterone) as a promising treatment for CRPC, even beyond second-generation AR-targeted therapies. Preclinical and clinical evidence have supported the use of supraphysiological testosterone to inhibit the growth of CRPC, and more recently abiraterone- and enzalutamide-resistant PC (4, 5). Despite encouraging efficacy in a subset of patients, treatment resistance develops. One pressing need on clinically managing CRPC is to identify response vs. resistance phenotypes to inform patients who will benefit from a treatment, and determine next line of therapy. While targeting AR is still under investigation in CRPC, Feng and He discuss the different AR dependent and independent paths to CRPC, and current pre-clinical and clinical developments aimed at mitigating disease progression. This review highlights advances and potential new opportunities for therapeutic intervention, including targeting different nodes of the AR signaling pathway, PARP inhibitors, and immunotherapy.

Although immunotherapy has not yet reached standard-of-care in CRPC, some patients respond exceptionally well. Ipilimumab is a human monoclonal antibody that binds to cytotoxic T-lymphocyte antigen 4 (CTLA-4). It blocks inhibitory signals expressed on activated T-cells and promotes anti-tumor activity (6). Phase III studies in PC have shown improved progression-free survival in some patients, albeit without an overall survival benefit (7, 8). Graff et al. identified 10 patients with metastatic PC with an incomplete response to ADT, and showed that three patients receiving ipilimumab achieved >50% PSA reduction with one patient achieving >90% reduction in PSA. Responders had an increase in effector memory T-cell subsets in blood and an increase in T-cell expression of T-bet, suggesting induction of a Th1 response. This study provides further rationale for future studies to identify a subset of CRPC patients who may respond to ipilimumab.

To identify new leads for CRPC treatment, Kim et al. used computational drug repositioning methods to repurpose existing drugs. The authors computationally integrated publicly available gene expression data of clinical CRPC, drug-induced gene expression data, and drug response data to determine key transcriptional perturbations in CRPC, then derived a computational reversal gene expression model to nominate drugs. Hence, they identified CRPC-associated genes MYL9, E2F2, APOE, and ZFP36 to be potentially reversed by existing drugs including sorafenib, olaparib, elesclomol, tanespimycin, and ponatinib. Importantly, lenalidomide combined with pazopanib was predicted to be the most potent therapy for CRPC.

CRPC continues to biologically evolve on treatment. With the appreciation of diverse genomic and molecular events driving CRPC progression, a multi-omics approach will be critical to define emerging CRPC phenotypes to predict therapeutic response and devise novel therapies.

## Author Contributions

H-ML, QC, and RdL wrote the manuscript. All authors approved it for submission.

## Conflict of Interest

The authors declare that the research was conducted in the absence of any commercial or financial relationships that could be construed as a potential conflict of interest.
